# Vital sign assessment and nursing interventions in medical and surgical patients with rapid response system triggers

**DOI:** 10.1111/jocn.16810

**Published:** 2023-06-27

**Authors:** Julie Considine, Alison M. Hutchinson, Imogen Mitchell, Shalika Bohingamu Mudiyanselage, Mohammadreza Mohebbi, Jennifer J. Watts, Tracey Bucknall

**Affiliations:** ^1^ School of Nursing and Midwifery and Centre for Quality and Patient Safety Research in the Institute for Health Transformation Deakin University Geelong Australia; ^2^ Centre for Quality and Patient Safety Research – Eastern Health Partnership Box Hill Australia; ^3^ Centre for Quality and Patient Safety Research – Barwon Health Partnership Geelong Australia; ^4^ Research and Academic Partnerships Canberra Health Services Canberra Australian Capital Territory Australia; ^5^ Australian National University College of Health and Medicine Canberra Australian Capital Territory Australia; ^6^ School of Health and Social Development, Deakin Health Economics, Institute for Health Transformation, Faculty of Health Deakin University Geelong Australia; ^7^ Biostatistics Unit, Faculty of Health Deakin University Burwood Victoria Australia; ^8^ Centre for Quality and Patient Safety Research – Alfred Health Partnership Melbourne Australia

**Keywords:** clinical decision‐making, clinical deterioration, nursing, patient assessment, rapid response system, vital signs

## Abstract

**Aim(s):**

To explore vital sign assessment (both complete and incomplete sets of vital signs), and escalation of care per policy and nursing interventions in response to clinical deterioration.

**Design:**

This cohort study is a secondary analysis of data from the Prioritising Responses of Nurses To deteriorating patient Observations cluster randomised controlled trial of a facilitation intervention on nurses' vital sign measurement and escalation of care for deteriorating patients.

**Methods:**

The study was conducted in 36 wards at four metropolitan hospitals in Victoria, Australia. Medical records of all included patients from the study wards during three randomly selected 24‐h periods within the same week were audited at three time points: pre‐intervention (June 2016), and at 6 (December 2016) and 12 months (June 1017) post‐intervention. Descriptive statistics were used to summarise the study data, and relationships between variables were examined using chi‐square test.

**Results:**

A total of 10,383 audits were conducted. At least one vital sign measurement was documented every 8 h in 91.6% of audits, and a complete set of vital signs was documented every 8 h in 83.1% of audits. There were pre‐Medical Emergency Team, Medical Emergency Team or Cardiac Arrest Team triggers in 25.8% of audits. When triggers were present, a rapid response system call occurred in 26.8% of audits. There were 1350 documented nursing interventions in audits with pre‐Medical Emergency Team (*n* = 2403) or Medical Emergency Team triggers (*n* = 273). One or more nursing interventions were documented in 29.5% of audits with pre‐Medical Emergency Team triggers and 63.7% of audits with Medical Emergency Team triggers.

**Conclusion:**

When rapid response system triggers were documented, there were gaps in escalation of care per policy; however, nurses undertook a range of interventions within their scope of practice in response to clinical deterioration.

**Relevance to Clinical Practice:**

Medical and surgical ward nurses in acute care wards frequently engage in vital sign assessment. Interventions by medical and nurgical nurses may occur prior to, or in parallel with calling the rapid response system. Nursing interventions are a key but under‐recognised element of the organisational response to deteriorating patients.

**Implications for the profession and/or patient care:**

Nurses engage in a range of nursing interventions to manage deteriorating patients, (aside from rapid response system activation) that are not well understood, nor well described in the literature to date.

**Impact:**

- This study addresses the gap in the literature regarding nurses' management of deteriorating patients within their scope of practice (aside from RRS activation) in real world settings.
- When rapid response system triggers were documented, there were gaps in escalation of care per policy; however, nurses undertook a range of interventions within their scope of practice in response to clinical deterioration.
- The results of this research are relevant to nurses working on medical and surgical wards.

**Reporting Method:**

The trial was reported according to the Consolidated Standards of Reporting Trials extension for Cluster Trials recommendations, and this paper is reported according to the Strengthening the Reporting of Observational Studies in Epidemiology Statement.

**Patient or Public Contribution:**

No Patient or Public Contribution.

**What does this paper contribute to the wider global clinical community?**

- This study highlights the important role of nurses in the management of deteriorating patients within their scope of practice.
- Nurses engage in a range of nursing interventions to manage deteriorating patients (aside from rapid response system activation) that are not well understood, nor well described in the literature to date.

## 1 INTRODUCTION

Recognising and responding to clinical deterioration is a core nursing responsibility. In Australia, recognising and responding to clinical deterioration in acute care hospitals is informed by two key documents. First, the National Consensus Statement (ACSQHC, 2010) describes the essential elements for timely recognition of, and response to, deteriorating patients, and second, the National Safety and Quality Health Service Standards (ACSQHC, 2011) required for hospital accreditation, in particular, relating to recognising and responding to acute clinical deterioration. Rapid response systems (RRSs) are a core patient safety strategy aimed at facilitating recognition of deteriorating patients, and escalation of care so that clinical deterioration can be managed at the point of care. As nurses have the most direct contact time with patients, they are commonly the first clinician to recognise clinical deterioration (Considine & Currey, 2015). Nurses are also the highest RRS users so the success of hospital RRSs is highly dependent on nurses' accuracy of patient assessment, interpretation of data and escalation of care when clinical deterioration is identified (Considine & Currey, 2015).

There are numerous published studies of vital sign assessment by nurses (Brekke et al., 2019; Bucknall et al., 2022; Mok, Wang, & Liaw, 2015) and nurses' compliance with RRS activation in patients with vital sign abnormalities (Bingham et al., 2015; Bucknall et al., 2022; Bucknall, Jones, Bellomo, Staples, & for the RESCUE Investigators, 2013; Guinane et al., 2013; Shearer et al., 2012). One of the largest studies was the Prioritising Responses of Nurses To deteriorating patient Observations (PRONTO) pragmatic cluster randomised controlled trial that tested the effect of a facilitation intervention on nurses' vital sign measurement, interpretation, and escalation of care for deteriorating patients (Bucknall et al., 2022).

The outcomes of interest were as follows: (i) vital sign assessments at least 8‐hourly on every patient; (ii) escalation of care per policy for patients with vital sign abnormalities; and (iii) implementation of appropriate nursing interventions in response to clinical deterioration (ACSQHC 2010). The control wards (*n* = 18) received standard dissemination of hospital policy requirements to achieve the outcomes of interest. This comprised distribution of the National Consensus Statement (ACSQHC 2010) and relevant National Safety and Quality Health Service Standards (ACSQHC 2011) by the Nurse Managers at staff meetings and via email, and notification of free online educational courses about recognition and response to deteriorating patients (Bucknall et al., 2017). The intervention wards (*n* = 18) had an external facilitator across hospitals, an internal hospital facilitator (HFLIP) and two ward facilitators (WFLIPs) per ward for 6 months who used facilitation methods to address barriers and leverage enablers to achieve the desired outcomes (Bucknall et al., 2022). There were no significant differences in any of the three outcomes of interest between intervention and control wards at 6 or 12 months post‐intervention (Bucknall et al., 2022).

It may be argued that the focus on nurses' compliance with vital sign measurement and RRS activation in patients with vital sign abnormalities is a simplistic view of nursing practice. It is possible that the nature of deterioration (such as the specific RRS trigger breached and the degree of abnormality) and nursing interventions for the management of deteriorating patients influence whether nurses do or do not activate the RRS. Whilst there are a number of studies detailing the interventions undertaken by the rapid response team (Flabouris et al., 2010; Jung et al., 2016; Mullins & Psirides, 2016; Silva et al., 2016; White et al., 2016), how nurses manage deteriorating patients within their scope of practice (aside from RRS activation) in real world settings is poorly understood.

### 1.1 Aim

The aims of this study were to explore: (i) the type and frequency of vital sign assessment (both complete and incomplete sets of vital signs); (ii) the relationship between escalation of care per policy and specific RRS triggers; and (iii) the relationship between escalation of care per policy and nursing interventions in response to clinical deterioration.

For the purpose of this study, escalation of care per policy was defined as escalation of care to the pre‐Medical Emergency Team (pre‐MET), Medical Emergency Team (MET) or Cardiac Arrest Team (CAT) in response to pre‐determined organisational criteria for each RRS tier (pre‐MET call for pre‐MET criteria, MET call for MET criteria and CAT call for cardiac arrest; Table S1). A complete set of vital signs was defined as documentation of respiratory rate, oxygen saturation, heart rate, blood pressure, conscious state and temperature (ACSQHC, 2010). Clinician concern was defined as care having been escalated without documented vital sign abnormalities.

## 2 METHODS

### 2.1 Design

This cohort study was a secondary analysis of data from the PRONTO cluster randomised controlled trial, the methodology and results of which are reported elsewhere [references redacted]. The trial was reported according to the Consolidated Standards of Reporting Trials extension for Cluster Trials (CONSORT) recommendations (Campbell et al., 2012), and this paper is reported according to the Strengthening the Reporting of Observational Studies in Epidemiology (STROBE) Statement (von Elm et al., 2007) (Table S2). This study was approved by Consultative Council for Clinical Trial Research (CCCTR) Victorian Streamlined Ethical Review Process (SERP) [HREC/16/ALFRED/25] and Deakin University [2016–248] (Bucknall et al., 2022).

### 2.2 Setting

The study was conducted in 36 wards at four university‐affiliated, metropolitan hospitals in Victoria, Australia, each with over 400 beds and providing acute and specialist services (Bucknall et al., 2017; Bucknall et al., 2022). The wards were a mix of medical and surgical wards comprising 21–46 beds per ward. The characteristics of the included wards are shown in Table S3. None of the study hospitals had an electronic medical record in place.

All four hospitals had a three‐tier RRS consisting of pre‐MET (single trigger), MET (single trigger) and CAT (Table S1). The CAT and MET were medically‐led teams of intensive care unit (ICU) clinicians who responded to deteriorating ward‐based patients, whilst the pre‐MET response was by ward‐based doctors and senior ward nurses. In addition, all four hospitals had paper‐based, colour coded observation and response charts that provided nurses with a visual prompt and guidance in terms of the appropriate organisational response, when documenting vital signs fulfilling pre‐MET or MET triggers. Nurse‐to‐patient ratios on all wards were 1:4 on morning and afternoon shifts and 1:8 overnight. Nurse staffing was a combination of registered nurses (Bachelor‐prepared) and enrolled nurses (Diploma‐prepared, working under the supervision of registered nurses).

### 2.3 Sample

All inpatients from the PRONTO study wards (medical and surgical) during the data collection periods were included. Patients from critical care, emergency, paediatrics, maternity, perioperative and psychiatric areas were excluded because these areas used alternative response systems for deteriorating patients. All study wards were subject to the same RRS criteria and governance, and nursing scope of practice; thus, the opportunities for escalation of care per policy and nursing interventions for deteriorating patients were the same for all patients.

### 2.4 Data collection

Medical record audits were conducted on all included patients at three time points (T0, T1, T2): pre‐intervention (T0), and at 6 (T1) and 12 months (T2) post‐intervention. Data were collected during three randomly selected 24‐hour periods within the same week in June 2016 (T0), December 2016 (T1) and June 2017 (T2); therefore, a single patient could be represented on multiple audit days (Bucknall et al., 2022). An electronic Case Report Form (eCRF) was used to collect study data. At each audit, documented vital sign data were collected: for vital signs fulfilling organisational pre‐MET and MET triggers (tailored to each organisation as per Table S1), data relating to any escalation of care per hospital policy were also collected. Data were collected by research assistants who received specific training on the electronic data collection tool and accompanying data dictionary. Inter‐rater reliability testing was established by independent double auditing across sites until Kappa >0.95 was achieved, with a minimum of 10 medical records audited per research assistant. In addition, project manager who was a registered nurse conducted study monitoring on a random sample of 100 patient charts across four sites to ensure data accuracy (Bucknall et al., 2017).

Fifteen nursing interventions were nominated apriori by the research team based on their clinical nursing expertise, hospital policies and nursing scope of practice. The apriori interventions were respiratory assessment, adjust oxygen therapy, reposition patient, withhold medication, administer medications as ordered, electrocardiography, encourage oral fluids, heated blankets, cardiovascular assessment, Glasgow Coma Score assessment, pain assessment, commence fluid balance chart, assess drainage fluid, reassess vital signs and other. If ‘other’ was selected, the research assistants were to add free text detail. Analysis of free text data by one nursing researcher (JC) resulted in six additional interventions based on frequencies: blood cultures (*n* = 31), deep breathing and coughing (*n* = 29), measure blood glucose level (*n* = 29), notify medical staff (*n* = 11), intravenous fluids (*n* = 6) and pathology testing (*n* = 5). The codes were ratified by the chief investigator (TB): examples of the free text coding process are shown in Table S4.

### 2.5 Data analysis

Data were analysed using SPSS Version 29.0. Descriptive statistics were used to summarise the study data. As data were not normally distributed (according to the Kolmogorov–Smirnov test), medians and interquartile ranges (IQRs) are presented. Relationships between variables were examined using chi‐square test, and statistical significance was indicated by *p* < 0.05. False‐discovery rate (FDR) was corrected by using the Benjamini–Hochberg approach (Benjamini & Hochberg, 1995) to mitigate the risk of type‐I error inflation from multiple comparisons; therefore, the cut‐off for statistical significance was more conservative than the usual 0.05. Pre‐MET and MET triggers were categorised as present or not present according to organisational thresholds detailed in Table S1.

## 3 RESULTS

A total of 10,383 audits were conducted across 3 days in a single week in June 2016 (*n* = 3370), December 2016 (*n* = 3535) and June 2017 (*n* = 3478). As there were the same patients (*n* = 6065) present during multiple audits, results are presented at the audit level rather than the patient level.

### 3.1 Vital sign assessment

Of the 10,383 audits, at least one vital sign measurement was documented every 8 h in 91.6% of audits (*n* = 9514) and a complete set of vital signs was documented every 8 h in 83.1% (*n* = 8631) of audits. In 86.8% of audits (*n* = 9015), there was evidence of further vital sign assessments in addition to the mandated 8‐hourly assessments: the median number of extra vital sign assessments was 2 (IQR = 1–3). The most frequently missing vital signs were temperature (2.7%, *n* = 279) and conscious state (2.7%, *n* = 278). The remaining vital signs had few missing values: respiratory rate (1.4%, *n* = 150), systolic blood pressure (1.2%, *n* = 121), heart rate (1.1%, *n* = 119) and oxygen saturation (1.1%, *n* = 116).

### 3.2 Escalation of care per policy in audits with RRS triggers

Of the 10,383 audits, 25.8% (*n* = 2680) had evidence of pre‐MET, MET or CAT triggers (pre‐MET 23.1%, *n* = 2403; MET 2.6%, *n* = 273 and CAT 0.3%, *n* = 4). When RRS triggers were present, a documented call to the appropriate tier of the RRS was evident in 26.8% of audits (Table 1).

**TABLE 1:** Frequency of rapid response system triggers and escalation of care per policy.

Rapid response system tier	Fulfilled RRS triggers	No escalation of care per policy		Escalation of care per policy	
	*n*	*n*	%	*n*	%
Pre‐MET	2403	1771	73.7	632	26.3
MET	273	186	68.1	87	31.9
CAT	4	0	0	4	100
**Total**	**2680**	**1957**	**73.2**	**723**	**26.8**

When pre‐MET triggers were present (*n* = 2403 audits), escalation of care per policy (pre‐MET call) was significantly *more likely* when hypotension (23.3% vs. 13.3%, FDR < 0.05), hyperthermia (18.5% vs. 3.1%, FDR < 0.05), hypertension (17.1% vs. 6.2%, FDR < 0.05), tachypnoea (9.8% vs. 5.1%, FDR < 0.05) and clinical concern (8.1% vs. 0.2%, FDR < 0.05) were present. Pre‐MET escalation of care was significantly *less likely* when there was hypoxaemia (7.1% vs. 16.4%, FDR < 0.05), bradycardia (4.9% vs. 7.9%, FDR = 0.012), decreased conscious state (3.5% vs. 16.4%, FDR < 0.05) and hypothermia (3.3% vs. 15.4%, FDR < 0.05) (Table 2). Three audits with documented clinical concern and no escalation of care also had documented hypotension (*n* = 2) or bradycardia (*n* = 1). Of the 51 audits with documented clinical concern and pre‐MET escalation of care, 50 had no documented vital sign abnormalities and one had documented tachycardia. Examples of pre‐MET clinical concern were decreased breath sounds, agitation, chest pain, epistaxis, family concern, falls and rectal bleeding.

**TABLE 2:** Escalation of care per policy versus pre‐MET or MET triggers.

Audits with pre‐MET triggers	Escalation of care per policy						
	No pre‐MET call (*n* = 1771)		Pre‐MET call (*n* = 632)		*p* ^a^ ^,^ ^c^	OR	95% CI
	n	%	n	%			
Patient characteristics							
Female gender	838	47.3	296	46.6	0.835^b^	0.981	0.81–1.17
Limitation of medical treatment orders	563	31.8	176	27.8	0.064	0.827	0.68–1.01
Emergency admission (vs. elective)	1559	88.1	530	83.9	0.007	0.703	0.54–0.91
Specific triggers							
Bradypnoea	16	0.9	4	0.6	0.520	0.698	0.23–2.01
Tachypnoea	90	5.1	62	9.8	<0.001	1.913	1.36–2.68
Hypoxaemia	290	16.4	45	7.1	<0.001	0.391	0.28–0.54
Bradycardia	140	7.9	31	4.9	0.012	0.601	0.40–0.89
Tachycardia	327	18.5	116	18.4	0.760	0.964	0.76–1.22
Hypertension	110	6.2	108	17.1	<0.001	3.110	2.34–4.13
Hypotension	235	13.3	147	23.3	<0.001	1.785	0.42–2.24
Decreased conscious state	291	16.4	22	3.5	<0.001	0.183	0.12–0.28
Hyperthermia	55	3.1	117	18.5	<0.001	6.958	4.98–9.71
Hypothermia	273	15.4	21	3.3	<0.001	0.188	0.12–0.29
Clinical concern	3	0.2	51	8.1	<0.001	51.702	16.07–166.28
Audits with MET triggers	No MET call (*n* = 186)		MET call (*n* = 87)		*p* ^a^ ^,c^		
	*n*	%	*n*	%			
Patient characteristics							
Female gender	96	51.6	38	43.7	0.222	0.727	0.46–1.21
Limitation of medical treatment orders	67	60.4	44	39.6	0.023	1.817	1.08–3.04
Emergency admission (vs. elective)	132	68.6	74	31.4	0.569	0.808	0.38–1.68
Specific triggers							
Tachypnoea	20	10.8	9	10.3	0.816	0.907	0.39–2.07
Hypoxaemia	53	28.5	15	17.2	0.045^c^	0.523	0.27–0.99
Bradycardia	8	4.3	0	0.0	0.058^b^	0.672	0.62–0.73
Tachycardia	17	9.1	24	27.6	<0.001	3.787	1.91–7.51
Hypertension	0	0.0	2	2.3	0.101^b^	0.314	0.26–0.37
Hypotension	79	42.5	22	25.3	0.006	0.458	0.26–0.81
Decreased conscious state	9	4.8	12	13.8	0.014	3.147	1.27–7.78
Clinical concern	0	0.0	10	11.5	<0.001^b^	24.02	3.02–190.92

OR, odd ratio; CI, confidence interval.

^a^ Chi‐square.

^b^ Fisher's exact test.

^c^ *p*‐values <0.03 were considered statistically significant to keep false discovery rate (FDR) <0.05 (Benjamini–Hochberg approach).

Of the audits with MET triggers (*n* = 273), escalation of care per policy (MET call) was significantly *higher* when tachycardia (27.6% vs. 9.1%, FDR < 0.05), decreased conscious state (13.8% vs. 4.8%, FDR = 0.014) and clinical concern (11.5% vs. 0.5%, FDR < 0.05) were present and significantly *lower* in the presence of hypotension (42.5% vs. 25.3%, FDR = 0.007) (hypoxaemia did not retain statistical significance following Benjamini–Hochberg correction). Of the ten audits with documented clinical concern and MET escalation of care, three had clinical concern (chest pain) documented in addition to documented tachycardia, one had documented general concern, tachycardia and decreased conscious state, and six had no documented vital sign abnormalities but had documented concerns regarding falls, uncontrolled pain and agitation. Patients who had emergency (versus elective) admissions had significantly less escalation of care per policy if pre‐MET triggers were present (83.9% vs. 88.1, FDR = 0.007) (Table 2).

### 3.3 Escalation of care per policy and specific nursing interventions

There was no significant difference in nursing interventions for pre‐MET (51.0% vs. 49.0%, *p* = 0.788, OR = 1.024, 95%CI: 0.86–1.22) or MET triggers (59.2% vs. 67.8%, *p* = 0.140, OR = 0.689, 95%CI: 0.42–1.13) between intervention and control wards. Overall, there were 1350 documented nursing interventions in audits with pre‐MET (*n* = 2403) or MET triggers (*n* = 273) (Table 3).

**TABLE 3:** Nursing interventions associated with presence of pre‐MET or MET triggers versus escalation of care per policy.

Documented nursing interventions	Presence of pre‐MET triggers (*n* = 2403 audits)									Presence of MET triggers (*n* = 273 audits)								
	Total number of nursing interventions (*n* = 1034)		No pre‐MET call (*n* = 1771)		Pre‐MET call (*n* = 632)		*p* ^a^ ^,^ ^c^	OR	95% CI	Total number of nursing interventions (*n* = 316)		No MET call (*n* = 186)		MET call (*n* = 87)		*p* ^a^ ^,^ ^d^	OR	95% CI
	*n*	%	*n*	%	*n*	%				*n*	%	*n*	%	*n*	%			
Administer medications as ordered^e^	209	20.2	69	3.9	140	22.2	<0.001	7.015	5.17–9.52	14	4.4	8	4.3	6	6.9	0.385^e^	1.648	0.54–4.90
Adjust oxygen therapy	162	15.7	126	7.1	36	5.7	0.221	0.788	0.24–1.15	52	16.5	39	21.0	13	14.9	0.237	0.662	0.33–1.32
Other^f^	158	15.3	80	4.5	78	12.3	<0.001	2.974	2.14–4.12	55	17.4	23	12.4	32	36.8	<0.001	4.123	2.22–7.64
Reassess vital signs	120	11.6	73	4.1	47	7.4	0.001	1.86	1.28–2.72	36	11.4	27	14.5	9	10.3	0.343	0.679	0.30–1.51
Perform electrocardiography	85	8.2	7	0.4	78	12.3	<0.001	35.460	16.27–77.29	36	11.4	10	5.4	26	29.9	<0.001	7.50	3.42–16.45
GCS assessment	67	6.5	54	3.0	13	2.1	0.193	0.667	0.36–1.23	29	9.2	2	1.1	27	31.0	<0.001	41.4	9.56–179‐26
Administer oral fluids	63	6.1	21	1.2	42	6.6	<0.001	5.929	3.48–10.09	18	5.7	13	7.0	5	5.7	0.700	0.811	0.28–2.35
Reposition patient	36	3.5	18	1.0	18	2.8	0.001	2.853	4.17–5.52	25	7.9	16	8.6	9	10.3	0.642	1.226	0.25–2.89
Blood cultures	30	2.9	7	0.4	23	3.6	<0.001	9.517	4.06–22.29	1	0.3	1	0.5	0	0.0	1.000^b^	N/A	N/A
Respiratory assessment	27	2.6	20	1.1	7	1.1	0.964	0.980	0.41–2.33	3	0.9	3	1.6	0	0.0	0.554^b^	N/A	N/A
Deep breathing & coughing	24	2.3	20	1.1	4	0.6	0.281	0.558	0.19–1.64	5	1.6	5	2.7	0	0.0	0.181^b^	N/A	N/A
Withhold medication	11	1.1	2	0.1	9	1.4	<0.001^b^	12.770	2.75–59.27	4	1.3	4	2.2	0	0.0	0.310^b^	N/A	N/A
Notify medical staff	8	0.8	2	0.1	6	0.9	0.005^b^	8.478	1.70–42.11	3	0.9	2	1.1	1	1.1	1.000^b^	1.070	0.09–11.96
Cardiovascular assessment	7	0.7	2	0.1	5	0.8	0.016^b^	7.049	1.36–36.28	2	0.6	1	0.5	1	1.1	0.537^b^	2.15	0.13–34.79
IV fluids	4	0.4	1	0.1	3	0.5	0.058^b^	8.442	0.88–81.30	2	0.6	2	1.1	0	0.0	1.000	N/A	N/A
Pain assessment	4	0.4	2	0.1	2	0.3	0.284^b^	2.806	0.39–19.96	1	0.3	0	0.0	1	1.1	0.319^b^	N/A	N/A
Pathology testing	4	0.4	1	0.1	3	0.5	0.058^b^	8.442	0.87–81.30	1	0.3	1	0.5	0	0.0	1.000^b^	0.680	0.63–0.74
Measure blood glucose level	3	0.3	0	0.0	3	0.5	0.018^b^	N/A	N/A	26	8.2	2	1.1	24	27.6	<0.001	35.048	8.05–152‐52
Assess drainage fluid	1	0.1	1	0.1	0	0.0	1.000^b^	N/A	N/A	3	0.9	2	1.1	1	1.1	1.000^b^	1.070	0.09–11.96
Commence fluid balance chart	1	0.1	0	0.0	1	0.2	0.263^b^	N/A	N/A	0	0.0	0	0.0	0	0.0	N/A	N/A	N/A
Provide heated blankets	10	1.0	7	0.4	3	0.5	0.729^b^	1.201	0.31–4.66	0	0.0	0	0.0	0	0.0	N/A	N/A	N/A

Abbreviations: OR, odd ratio CI = confidence interval.

^a^ Chi‐square.

^b^ Fisher's exact test.

^c^ *p*‐values <0.02 were considered statistically significant to keep false discovery rate (FDR) < 0.05 (Benjamini–Hochberg approach).

^d^ *p*‐values <0.01 were considered statistically significant to keep false discovery rate (FDR) <0.05 (Benjamini–Hochberg approach).

^e^ Included pro re nata (prn), immediate single dose (stat) or adjusted timing of patient's ordered medications.

^f^ Included cold compress, commencement of alcohol withdrawal scale, suction, commencement of visual acuity chart, patient education.

#### 3.3.1 Audits with pre‐MET triggers

One or more nursing interventions were documented in 29.5% (*n* = 710/2403) of audits with pre‐MET triggers. The median number of nursing interventions in audits with pre‐MET triggers was 1 (IQR = 1–2). The most common nursing interventions associated with pre‐MET triggers were medication administration (20.2%), adjustment to oxygen therapy (15.7%) and vital sign reassessment (11.6%). Nursing interventions were significantly *more likely* when there were pre‐MET triggers and care was escalated per policy (55.1%, *n* = 348/632) compared to pre‐MET triggers and no care escalation (24.0%, *n* = 362/ 1771) (*p* < 0.001). When pre‐MET triggers were present, the following nursing interventions were significantly *more likely* when care was escalated per policy: medication administration, vital sign reassessment, electrocardiography, oral fluid administration, patient repositioning, blood culture collection, withholding of medication, medical staff notification, cardiovascular assessment and blood glucose measurement (Table 3).

#### 3.3.2 Audits with MET triggers

One or more nursing interventions were documented in 63.7% (*n* = 174/273) of audits with MET triggers. The median number of nursing interventions in audits with MET triggers was 1 (IQR = 1–3). The most common nursing interventions associated with MET triggers were adjustment to oxygen therapy (16.5%), electrocardiography (11.4%) and vital sign reassessment (11.4%). Nursing interventions were significantly *more likely* when there were MET triggers and care was escalated per policy (78.2%, *n* = 68/87) compared to MET triggers and no care escalation (57.0%, *n* = 106/186) (*p* < 0.001). When MET triggers were present, the following nursing interventions were significantly *more likely* when care was escalated per policy: electrocardiography, Glasgow Coma Score assessment and blood glucose measurement (Table 3).

## 4 DISCUSSION

This study had four major findings: there were high levels of 8‐hourly vital sign assessment and documentation of complete sets of vital signs as per policy; there was low compliance with escalation of care per policy in the presence of pre‐MET, MET and CAT triggers; specific pre‐MET and MET triggers influenced whether care was escalated per policy; and nursing interventions were common when pre‐MET or MET triggers were present. These major findings will be discussed in the sections to follow.

First, 91.6% of audits had at least one vital sign assessment every 8 h and 83.1% of audits had a complete set of vital signs documented every 8 h. Our findings are different to those of other studies that report proportions of vital signs assessments with one or more missing vital signs ranging from zero to 41% (Bleyer et al., 2011; Cahill et al., 2011; Chen et al., 2009). The implementation of national standards for frequency of vital sign assessment, making 8‐hourly vital sign assessment a minimum standard for hospital accreditation, use of human factors in the design of observation charts, and educational interventions aimed at increasing recognition of clinical deterioration and RRS activation, may be possible explanations for this finding (ACSQHC, 2010, 2011). The most frequently missing vital signs in our study were temperature (2.7%, *n* = 279) and conscious state (2.7%, *n* = 278).

Bleyer et al. (2011) also reported temperature as the most common missing vital sign, albeit at a much higher rate (17.8%) than in our study. Acknowledging there were low levels of missing temperature documentation in our study, it may be hypothesised that the need to engage in increased activity (e.g. sourcing a tympanic thermometer) negatively influenced measurement and documentation of temperature. A number of authors report respiratory rate as the least documented vital sign (Cahill et al., 2011; Cardona‐Morrell et al., 2016; Chen et al., 2009), however, in our study, only 1.4% of audits were missing documentation of respiratory rate. The importance of respiratory rate as a sensitive and specific vital sign indicator of serious illness has gained increased recognition in the decade prior to the trial (Cretikos et al., 2008), which may in part have influenced this finding.

Second, escalation of care per policy occurred in only one in four audits with pre‐MET, MET and CAT triggers. Pre‐MET, MET and CAT triggers were present in 25.8% of audits in this study, and pre‐MET triggers were more common than MET triggers (23.1% vs 2.6%). Failure to escalate care per policy occurred in 73.7% of audits with pre‐MET triggers and 68.1% of audits with MET triggers. The majority of studies to date have focused on either pre‐MET or MET RRS tiers; there are few published studies to date that examine the pre‐MET and MET RRS tiers as a continuum within the same patients (Bingham et al., 2015; Flabouris et al., 2015). Our findings are reflective of those of Bingham et al. (2015) and Flabouris et al. (2015) whose single‐site studies showed that pre‐MET triggers are more common than MET triggers, presumably because of the wider thresholds of pre‐MET triggers. In these studies, failure to escalate care per policy for pre‐MET and MET triggers were measured using different methods, limiting direct comparisons between studies. In 24‐h point prevalence studies, failure to escalate care per policy when pre‐MET or MET triggers were present ranged from 45.1% (47% pre‐MET and 30% MET triggers) to 50.9% (52.2% pre‐MET triggers and 45.5% MET triggers) (Bingham et al., 2015; Flabouris et al., 2015). At the patient level, failure to escalate care for pre‐MET or MET triggers during the course of hospital admission was reported in 51.6% of patients (65.0% pre‐MET and 35.3% MET, *p* < 0.01) (Flabouris et al., 2015). At the trigger level, 58.7% of pre‐MET or MET triggers were not escalated (64.7% pre‐MET and 40% MET, no *p* value reported) (Flabouris et al., 2015).

Whilst there were gaps in escalation of care per policy (pre‐MET call for pre‐MET triggers and MET call for MET triggers) in this study, it is possible that nurses used alternative methods of escalation of care to mobilise medical staff to the point of care. It is well documented that nurses commonly escalate care to treating (admitting) teams instead of activating the RRS (Jones et al., 2006; Shearer et al., 2012). Other studies have shown that nurses used the RRS if they perceived the treating team were not responding appropriately (Bingham et al., 2020; Jones et al., 2006) and it is also possible that nurses prefer to communicate their concerns to ward medical staff with whom they have a relationship over MET responders who they may not know.

Third, escalation of care per policy varied according to specific pre‐MET or MET triggers. Higher likelihood of escalation of care occurred in the presence of pre‐MET blood pressure derangements, hyperthermia and tachypnoea, and MET triggers tachycardia and decreased conscious state. Other studies also show altered conscious state and tachycardia are the most common MET triggers (Davies et al., 2014; Mullins & Psirides, 2016) and had zero failure to escalate rates (Flabouris et al., 2015). It is possible in our study that pre‐MET escalation of care for tachypnoea, may have resulted in early intervention and circumvented later MET calls for tachypnoea, which has two important implications. First, it may be proposed that nurses are now recognising the importance of tachypnoea as an indicator of deterioration, which is supported by the low rates of missing documentation of respiratory rate (1.4%) in our study and that 87.5% of nurses in another study agreed that the respiratory rate criterion of the RRS had the most benefit (Davies et al., 2014). Second, the relationship between pre‐MET escalation of care and interventions in reducing physiological derangement to MET trigger levels warrants further research.

Of concern is failure to escalate care. Pre‐MET hypoxemia, bradycardia, decreased conscious state, and hypothermia, and MET level hypotension were all associated with lower likelihood of pre‐MET or MET call, respectively. There are few published papers that report pre‐MET failure to escalate rates; however, our findings resemble those of Flabouris et al. (2015) who reported bradycardia (75%) and hypothermia (66.7%) as having the highest failure to escalate rates of all pre‐MET triggers. MET activation failure in the presence of hypotension is reported in other studies and ranges from 32% to 39% (Considine et al., in press; Flabouris et al., 2015). Further, there is conflict in the literature about nurses' perceptions of the importance of blood pressure with as many as 60% of nurses in one study believing (erroneously) that blood pressure changes are the first indicator of clinical deterioration (Mok, Wang, Cooper, et al., 2015). Nurses in another study, however, ranked mean arterial pressure as the RRS trigger with the second lowest level of perceived benefit (Davies et al., 2014). In our study, there was decreased likelihood of escalation of care when pre‐MET and MET hypoxaemia were present; however, statistical significance did not remain for MET hypoxaemia following Benjamini–Hochberg correction. Failure to escalate care for MET hypoxemia ranged from 36% to 64.7% (Considine et al., in press; Flabouris et al., 2015), and in both of these studies, hypoxemia was the most common trigger associated with failure to activate the MET Again, there are conflicting reports in the literature with 60% of nurses relying on oxygen saturation to evaluate respiratory dysfunction (Mok, Wang, Cooper, et al., 2015) but nurses ranking oxygen saturation as the RRS triggers with the lowest perceived benefit (Davies et al., 2014). In our study, hyperthermia was associated with increased likelihood of escalation of care in both pre‐MET and MET contexts and this may be due to long‐standing initiatives such as the focus on early recognition and response to sepsis and improvements in processes of care for patients with sepsis. The reasons why some triggers are more or less likely to result in care escalation are unclear and the importance nurses place on specific vital signs as indicators of clinical deterioration should be a focus of future research.

Clinical concern was associated with increased likelihood of escalation of care in both pre‐MET and MET contexts. In our study, almost all of the pre‐MET escalations of care and 60% of MET escalations of care for clinical concern occurred in the absence of documented vital sign abnormalities. Nurses are the most frequent users of the clinician concern RRS criterion and clinician concern is a common reason for MET activation (Douw et al., 2015; Flabouris et al., 2015; Santiano et al., 2009; Silva et al., 2016) suggesting that nurses make conscious and deliberate decisions that a patient is deteriorating, even in the absence of MET triggers. A 2020 systematic review showing that nurses used 26 subjective and 147 objective cues to identify changes in patients' clinical states and 94% of the cues used were aimed at recognising clinical deterioration (Burdeu et al., 2021) reinforces the notion that recognition of deteriorating patients goes beyond vital signs. Further, it is well documented that nurse concern often precedes vital sign physiological changes in deteriorating patients and there is evidence that RRS activation for clinician concern improves patient outcomes (Douw et al., 2015; Santiano et al., 2009).

Finally, one third of audits with pre‐MET triggers and two thirds of audits with MET triggers had one or more documented nursing interventions. Further, nursing interventions were significantly more likely in audits where care was escalated per policy, suggesting nurses' response to deteriorating patients is more than simply escalating care using pre‐MET or MET pathways. Evidence of nurse‐initiated interventions for pre‐MET and MET triggers is consistent with nurses' responsibility to respond to deteriorating patients and mitigate risk (Considine & Currey, 2015; Massey et al., 2017) and reflects findings from other studies that show once clinical deterioration is recognised, nurses actively engage in interventions within their scope of practice (Bingham et al., 2020; Donohue & Endacott, 2010; Guinane et al., 2013).

The nursing management of deteriorating patients within nurses' scope of practice is under‐reported in the literature. Possible explanations for this paucity of literature may be under‐representation of nurses' roles in recognition and response to clinical deterioration at policy level (Considine et al., 2018; Sprogis et al., 2021), the high focus on nurses' failure to recognise clinical deterioration or lack of compliance with RRS activation (Bingham et al., 2015; Bucknall et al., 2013; Guinane et al., 2013; Shearer et al., 2012), or under‐documentation of nursing interventions. Whilst there are a number of studies detailing the interventions implemented by the rapid response team (Flabouris et al., 2010; Mullins & Psirides, 2016; Silva et al., 2016; White et al., 2016), how nurses manage deteriorating patients prior to RRS activation or whilst awaiting team arrival is a major knowledge gap worthy of investigation.

### 4.1 Limitations

The study data were collected using medical record audit so there is the possibility that documentation may not reflect clinical practice behaviours; however, medical records are a legal representation of the care delivered and are commonly used in studies of safety and quality of care. Further, this limitation was moderated by a multisite design and data abstraction performed by trained researchers using a comprehensive data dictionary to optimise data reliability. It is possible that patients with pre‐MET or MET triggers had their care escalated (either per policy or via alternative means) and their clinical deterioration managed appropriately but it was not reflected in the documentation. The relatively small numbers of escalations of care per policy and nursing interventions limited statistical analyses, particularly some of the sub‐analyses, so future studies with larger samples are warranted.

## 5 CONCLUSION

Medical and surgical ward nurses in acute care wards are meeting or exceeding national standards regarding vital sign assessments. When pre‐MET or MET triggers were documented, there were gaps in escalation of care per policy; however, nurses undertook a range of interventions within their scope of practice in response to clinical deterioration. It was beyond the scope of this study to examine the outcomes from nursing interventions for deteriorating patients in terms of resolution of pre‐MET or MET triggers and RRS activation, but this is an important area for future research. There were relationships between escalation of care per policy and specific pre‐MET and MET triggers: the importance that nurses give to specific physiological parameters and how different triggers impact on nurses' response to clinical deterioration and RRS activation warrants further investigation.

## AUTHOR CONTRIBUTIONS

**Considine:** Conceptualisation, Methodology, Validation, Formal Analysis, Investigation, Data curation, Writing—original draft, Supervision, Project administration, Funding acquisition **Hutchinson:** Conceptualisation, Methodology, Validation, Formal Analysis, Investigation, Data curation, Writing—original draft, Supervision, Project administration, Funding acquisition **Mitchell:** Conceptualisation, Methodology, Validation, Formal Analysis, Investigation, Data curation, Writing—original draft, Funding acquisition **Bohingamu Mudiyanselage:** Formal Analysis, Data curation, Writing—review & editing **Mohebbi:** Methodology, Validation, Formal Analysis, Investigation, Data curation, Writing—review & editing, Supervision, Project administration, Funding acquisition **Watts:** Methodology, Validation, Formal Analysis, Data curation, Writing—review & editing, Funding acquisition **Bucknall** Conceptualisation, Methodology, Validation, Formal Analysis, Investigation, Data curation, Writing—original draft, Supervision, Project administration, Funding acquisition.

## FUNDING INFORMATION

The trial was funded by the National Health and Medical Research Council and partner organisations: Alfred Health, Eastern Health, Monash Health, Australian Commission on Safety and Quality in Health Care and SA Health. National Health and Medical Research Council Partnership Project Grant ID1114545.

## CONFLICT OF INTEREST STATEMENT

The authors have no conflicts of interest to declare.

## STATISTICS

The authors have checked to make sure that our submission conforms as applicable to the Journal's statistical guidelines. There is a statistician on the author team (Mohebbi).

## Supporting information


**Table S1:** Rapid Response System criteria per study site
**Table S2:** STROBE Statement—Checklist of items that should be included in reports of cohort studies (von Elm et al., 2007)
**Table S3:** Study ward charactersitics
**Table S4:** Examples of recoding of ‘other’ nursing interventions from free text data

## Data Availability

The data that support the findings of this study are available on request from the corresponding author. The data are not publicly available due to privacy or ethical restrictions.
