# Supplementary material for: Vital sign assessment and nursing interventions in medical and surgical patients with rapid response system triggers
Source: J Clin Nurs. 2023 Jun 27;32(19-20):7310–20. doi: 10.1111/jocn.16810 (PMC10946594; doi:10.1111/jocn.16810)
Supplement: Supplementary file 1 — Table S1: Rapid Response System criteria per study site Table S2: STROBE Statement—Checklist of items that should be included in reports of cohort studies (von Elm et al., 2007) Table S3: Study ward charactersitics Table S4: Examples of recoding of ‘other’ nursing interventions from free text data [file JOCN-32-7310-s001.docx]

Supplementary Table 1: Rapid Response System criteria per study site

|  | Hospital A | Hospital B | Hospital C | Hospital D |
| --- | --- | --- | --- | --- |
| **Pre-Medical emergency team criteria** | | | | |
| Respiratory rate (breaths / minute) | 6-12 or  25-29 | 7-10 or  25-35 | 8-10 or  25-29 | 6-12 or  25-29 |
| Oxygen saturation (%) | 91-93 | 91-93 | 91-93 | 91-93 |
| Heart Rate (beats / minute) | 41-50 or  100-130 | 41-50 or  120-139 | 100-130 | 41-50 or  100-130 |
| Systolic blood pressure (mmHg) | 180-199 | 91-100 or  180-199 | 180-199 | 180-199 |
| Conscious state | Not a criterion at this site | Previously alert, now only  responsive to  verbal stimuli | Not a criterion at this site | Not a criterion at this site |
| Temperature | <36.0^0^C or >38.3^0^C | <36.0^0^C or >38.3^0^C | <36.0^0^C or >38.3^0^C | <36.0^0^C or >38.3^0^C |
| New or unrelenting chest pain | ✓ | ✓ | ✓ | ✓ |
| New or unrelenting shortness of breath | ✓ | Not a criterion at this site | ✓ | ✓ |
| Increased & unexpected blood or fluid loss | ✓ | Not a criterion at this site | ✓ | ✓ |
| Clinician concern | ✓ | Not a criterion at this site | ✓ | ✓ |
| **Medical emergency team criteria** | | | | |
| Airway | Threatened | Threatened | Threatened | Threatened |
| Respiratory rate (breaths / minute) | <6 or >30 | <6 or >36 | <8 or >30 | <6 or >30 |
| Oxygen saturation (%) | <90% on oxygen | <90% on oxygen | <90% on oxygen | <90% on oxygen |
| Heart Rate (beats / minute) | >130 | <40 or >140 | <50 or >130 | >130 |
| Systolic blood pressure (mmHg) | <90 or >200 | <90 or >200 | <90 | <90 or >200 |
| Conscious state | Any unexpected  decrease | Any unexpected  decrease  Fall in  GCS > 2points | Any unexpected  decrease | Any unexpected  decrease |
| Fitting | ✓ | ✓ | ✓ | ✓ |
| New or unrelenting chest pain | Not a criterion at this site | Not a criterion at this site | ✓ | Not a criterion at this site |
| Pain | Not a criterion at this site | Uncontrolled | Not a criterion at this site | Not a criterion at this site |
| Clinician concern | ✓ | ✓ | ✓ | ✓ |
| **Cardiac arrest criteria** | | | | |
| Cardiorespiratory arrest | ✓ | ✓ | ✓ | ✓ |
| Unresponsive | ✓ | ✓ | ✓ | ✓ |
|  | | | | |
| mmHg = millimeters of mercury | | | | |

Supplementary Table 2: STROBE Statement—Checklist of items that should be included in reports of cohort studies (von Elm et al., 2007)

|  | Item No | Recommendation | Page No |
| --- | --- | --- | --- |
| **Title and abstract** | 1 | (*a*) Indicate the study’s design with a commonly used term in the title or the abstract | 1-2 |
|  |  | (*b*) Provide in the abstract an informative and balanced summary of what was done and what was found | 1-2 |
| Introduction | | | |
| Background/rationale | 2 | Explain the scientific background and rationale for the investigation being reported | 5-7 |
| Objectives | 3 | State specific objectives, including any prespecified hypotheses | 7 |
| Methods | | | |
| Study design | 4 | Present key elements of study design early in the paper | 7-8 |
| Setting | 5 | Describe the setting, locations, and relevant dates, including periods of recruitment, exposure, follow-up, and data collection | 8 |
| Participants | 6 | (*a*) Give the eligibility criteria, and the sources and methods of selection of participants. Describe methods of follow-up | 9 |
|  |  | (*b*) For matched studies, give matching criteria and number of exposed and unexposed | N/A |
| Variables | 7 | Clearly define all outcomes, exposures, predictors, potential confounders, and effect modifiers. Give diagnostic criteria, if applicable | 9-10 |
| Data sources/ measurement | 8* | For each variable of interest, give sources of data and details of methods of assessment (measurement). Describe comparability of assessment methods if there is more than one group | 9-10 |
| Bias | 9 | Describe any efforts to address potential sources of bias | 9-10 |
| Study size | 10 | Explain how the study size was arrived at | 9-10 |
| Quantitative variables | 11 | Explain how quantitative variables were handled in the analyses. If applicable, describe which groupings were chosen and why | 9-10 |
| Statistical methods | 12 | (*a*) Describe all statistical methods, including those used to control for confounding | 10-11 |
|  |  | (*b*) Describe any methods used to examine subgroups and interactions | N/A |
|  |  | (*c*) Explain how missing data were addressed | N/A |
|  |  | (*d*) If applicable, explain how loss to follow-up was addressed | N/A |
|  |  | (*e*) Describe any sensitivity analyses | N/A |
| Results | | |  |
| Participants | 13* | (a) Report numbers of individuals at each stage of study—eg numbers potentially eligible, examined for eligibility, confirmed eligible, included in the study, completing follow-up, and analysed | 11 |
|  |  | (b) Give reasons for non-participation at each stage | N/A |
|  |  | (c) Consider use of a flow diagram | N/A |
| Descriptive data | 14* | (a) Give characteristics of study participants (eg demographic, clinical, social) and information on exposures and potential confounders | 11 |
|  |  | (b) Indicate number of participants with missing data for each variable of interest | N/A |
|  |  | (c) Summarise follow-up time (eg, average and total amount) |  |
| Outcome data | 15* | Report numbers of outcome events or summary measures over time | 11-14 |
| Main results | 16 | (*a*) Give unadjusted estimates and, if applicable, confounder-adjusted estimates and their precision (eg, 95% confidence interval). Make clear which confounders were adjusted for and why they were included | 11-14 |
|  |  | (*b*) Report category boundaries when continuous variables were categorized | N/A |
|  |  | (*c*) If relevant, consider translating estimates of relative risk into absolute risk for a meaningful time period | N/A |
| Other analyses | 17 | Report other analyses done—eg analyses of subgroups and interactions, and sensitivity analyses | N/A |
| Discussion | | | |
| Key results | 18 | Summarise key results with reference to study objectives | 14-19 |
| Limitations | 19 | Discuss limitations of the study, taking into account sources of potential bias or imprecision. Discuss both direction and magnitude of any potential bias | 20 |
| Interpretation | 20 | Give a cautious overall interpretation of results considering objectives, limitations, multiplicity of analyses, results from similar studies, and other relevant evidence | 20 |
| Generalisability | 21 | Discuss the generalisability (external validity) of the study results | N/A |
| Other information | | | |
| Funding | 22 | Give the source of funding and the role of the funders for the present study and, if applicable, for the original study on which the present article is based | Title page |

*Give information separately for exposed and unexposed groups.

**Note:** An Explanation and Elaboration article discusses each checklist item and gives methodological background and published examples of transparent reporting. The STROBE checklist is best used in conjunction with this article (freely available on the Web sites of PLoS Medicine at http://www.plosmedicine.org/, Annals of Internal Medicine at http://www.annals.org/, and Epidemiology at http://www.epidem.com/). Information on the STROBE Initiative is available at http://www.strobe-statement.org.

Supplementary Table 3: Study ward charactersitics

| **Wards** | **Bed numbers** |
| --- | --- |
| **Hospital A (n=10)** |  |
| - General medicine | 26 |
| - General medicine | 20 |
| - Nephology | 22 |
| - Respiratory, dermatology, endocrinology and infectious diseases | 26 |
| - Neuroscience, neurosurgery, stroke and neurology | 36 |
| - Acute assessment unit | 26 |
| - Cardiothoracic | 24 |
| - Upper gastrointestinal surgery | 22 |
| - General medicine, haematology, rheumatology | 26 |
| - Coronary care unit | 21 |
| **Hospital B (n=8)** |  |
| - Acute assessment unit | 24 |
| - Plastic surgery | 20 |
| - Orthopaedic surgery | 36 |
| - General medicine | 36 |
| - General surgical | 24 |
| - Gastroenterology, colorectal, vascular surgery and diabetic foot care | 20 |
| - Colorectal, orthopaedic, fast track & rehabilitation | 36 |
| - General medicine | 36 |
| **Hospital C (n=8)** |  |
| - Cardiology and respiratory | 32 |
| - General medicine | 32 |
| - Bariatric, colorectal, breast, and upper gastrointestinal surgery | 32 |
| - General surgical | 28 |
| - Haematology | 30 |
| - General medicine | 32 |
| - Gynaecological and ear, nose and throat surgery | 24 |
| - Neurology and gastroenterology | 28 |
| **Hospital D (n=10)** |  |
| - Trauma and orthopaedics | 46 |
| - Respiratory | 28 |
| - Neurosurgery | 36 |
| - General medicine | 32 |
| - Colorectal and upper gastrointestinal surgery | 30 |
| - Cardiology and cardiothoracics | 46 |
| - General medicine | 32 |
| - Plastic, ear, nose and throat surgery and burns | 29 |
| - General medicine | 34 |
| - Haematology and oncology | 34 |
|  |  |

Supplementary Table 4: Examples of recoding of ‘other’ nursing interventions from free text data

| **Free text in data set** | **New intervention code** |
| --- | --- |
| Bloods taken and BSL | Pathology testing |
| Bloods taken, Electrolytes replaced | Pathology testing |
| Blood test ordered, not specified what | Pathology testing |
| As a result of the review fluid bolus was administered | IV fluids |
| Increase IVT and blood cultures as per MO | IV fluids |
| IV fluids | IV fluids |
| Patient was being managed for post-op  blood loss. Blood transfusion and IVT given | IV fluids |
| Aays DB (deep breathing) on obs chart. SaO2 then recorded as being greater than 92%. | DB&C |
| DB+C encouraged. O2 Sats improved to 91%  Documented 15/6 - Aim O2 Sats > or equal to 90% | DB&C |
| Deep breathing and coughing | DB&C |
| paged cardiology to review regular medications | Notify medical staff |
| Contacted doctor for CR [clinical review] | Notify medical staff |
| CR [clinical review] team informed | Notify medical staff |
| Dr. paged | Notify medical staff |
| Dr. paged | Notify medical staff |
| Dr. paged. Stat order 25mcg GTN patch admin. | Notify medical staff |
| HM0 paged twice. | Notify medical staff |
| HMO notified | Notify medical staff |
| IVT started. Dr. paged. HR 111 | Notify medical staff |
| BP meds | Measure blood glucose level |
| BSL | Measure blood glucose level |
| 'Enc DB & C ++' | Measure blood glucose level |
| NG feeds stopped, BSL, suction | Measure blood glucose level |
| Attempted to collect Blood Cultures | Blood cultures |
| Attempted to take blood cultures but patient wasn't keen. | Blood cultures |
| Blood cultures | Blood cultures |
| Temp was 38.1 which although not in the yellow zone is considered febrile. Blood cultures taken | Blood cultures |
| Patient had a temp of 38.1. Not reportable but febrile, the tachycardia was treated as being connected to that.  Blood Cultures | Blood cultures |
